# Neural and contractile determinants of burst‐like explosive isometric contractions of the knee extensors

**DOI:** 10.1111/sms.14244

**Published:** 2022-11-16

**Authors:** Samuel D'Emanuele, Cantor Tarperi, Alberto Rainoldi, Federico Schena, Gennaro Boccia

**Affiliations:** ^1^ School of Sport and Exercise Sciences, Department of Neurosciences, Biomedicine and Movement Sciences University of Verona Verona Italy; ^2^ Neuromuscular Function Research Group, Department of Clinical and Biological Sciences University of Turin Turin Italy; ^3^ Neuromuscular Function Research Group, Department of Medical Sciences University of Turin Turin Italy

**Keywords:** explosive contractions, HDsEMG, muscle fiber conduction velocity, M‐wave, octets, peripheral nerve stimulation

## Abstract

Walking and running are based on rapid burst‐like muscle contractions. Burst‐like contractions generate a Gaussian‐shaped force profile, in which neuromuscular determinants have never been assessed. We investigated the neural and contractile determinants of the rate of force development (RFD) in burst‐like isometric knee extensions. Together with maximal voluntary force (MVF), voluntary and electrically evoked (8 stimuli at 300 Hz, octets) forces were measured in the first 50, 100, and 150 ms of burst‐like quadriceps contractions in 24 adults. High‐density surface electromyography (HDsEMG) was adopted to measure the root mean square (RMS) and muscle fiber conduction velocity (MFCV) from the vastus lateralis and medialis. The determinants of voluntary force at 50, 100, and 150 ms were assessed by stepwise multiple regression analysis. Force at 50 ms was explained by RMS (*R* ^2^ = 0.361); force at 100 ms was explained by octet (*R* ^2^ = 0.646); force at 150 ms was explained by MVF (*R* ^2^ = 0.711) and octet (*R* ^2^ = 0.061). Peak RFD (which occurred at 60 ± 10 ms from contraction onset) was explained by MVF (*R* ^2^ = 0.518) and by RMS_50_ (*R* ^2^ = 0.074). MFCV did not emerge as a determinant of RFD. Muscle excitation was the sole determinant of early RFD (50 ms), while contractile characteristics were more relevant for late RFD (≥100 ms). As peak RFD is mostly determined by MVF, it may not be more informative than MVF itself. Therefore, a time‐locked analysis of RFD provides more insights into the neuromuscular characteristics of explosive contractions.

## 1 INTRODUCTION

The rate of force development (RFD) reflects the ability to rapidly increase muscle force after the onset of a ballistic contraction.^1^, ^2^ The RFD of knee extensor muscles has been shown to be an important determinant of performance in explosive tasks such as vertical jumping,^3^ weightlifting,^4^ and cycling.^5^ In addition, the relevance of possessing a high RFD has also been demonstrated in locomotor tasks such as endurance running^6^ and in functional activities of daily living.^7^, ^8^ All the afore‐mentioned movements are indeed characterized by time‐constrained contractions, typically lasting less than 150–200 ms.

The intersubject variance in voluntary knee extensors' RFD can be partially explained by variance in MVF. However, the role of MVF is more pronounced in the late phase of the contraction, and it was found to be the primary determinant of RFD from 75 ms onwards.^9^, ^10^ The contribution of neural and contractile mechanisms varies throughout the time course of the force‐time curve rise. Indeed, the greater the time elapsed from contraction onset; the more muscular factors predominate on neural ones. Agonist muscle excitation, as measured by the EMG amplitude calculated over the first 50 ms from the contraction onset, is strongly correlated (*r* ≈ 0.7–0.8) to the RFD in the first 50 ms.^10^, ^11^, ^12^, ^13^ This was even more elegantly demonstrated by Del Vecchio et al,^14^ in the tibialis anterior, where the rates of motor unit recruitment and motor unit firing are the most influential factors associated with the RFD in the first 50 ms of contractions. Conversely, the quadriceps muscle thickness^11^ and volume^15^ are mostly correlated to the RFD in the later phases of the contraction (≥100 ms). The responses to electrically evoked octets (eight stimuli delivered at 300 Hz) have been used to evaluate the maximal contractile (involuntary) RFD.^13^, ^16^ Electrically evoked responses correlate to voluntary RFD throughout the whole contraction (Pearson's *r* ranging from 0.4 to 0.8 from 25 to 150 ms), with a stronger relation with later RFD (≥100 ms). Finally, RFD is also partially explained by muscle and tendon stiffness.^17^ However, the correlation between RFD and tendon stiffness was found to be no longer present when the effect of MVF was accounted for.^15^, ^18^

The timing and shape of force production during ballistic contractions is crucial. To elucidate the neuromuscular determinants of RFD, the above‐mentioned studies adopted ballistic contractions with various durations from ≈1 to 3 s. Indeed, some studies adopted explosive contractions lasting 3 s,^9^, ^11^ others explosive contractions lasting 1 s,^10^, ^15^, ^18^ and others did not mention the length of the contraction.^19^ Human locomotion has been described as being generated by an impulsive (burst‐like) excitation of muscle groups.^20^ The activation profiles are Gaussian‐shaped curves^21^, ^22^ without any holding phase. Requesting that participants maintain the muscle contraction for 1 s is an effective strategy to reach a high level of force (>70% of MVF), which is necessary to produce the maximum possible RFD. Nevertheless, the muscle activation profile of such a motor task does not reflect the ones adopted in locomotion. Therefore, we still do not have a clear understanding of RFD determinants in Gaussian‐shaped muscle activation profiles closer to locomotion requirements.^21^, ^22^

Two recent reviews investigating the effect of strength training^23^ and muscle fatigue^24^ on RFD reported that the most studied variable of RFD is the RFD_peak_, that is, the local maximum of the first derivative of force with respect to time. While RFD_peak_ is the most popular RFD‐related variable, the contractile and neural determinants of RFD_peak_ have never been investigated, as all previous studies focused on time‐locked RFD variables such as RFD in the first 50, 100, or 150 ms.

Therefore, we aimed to investigate the neural and contractile determinants of (1) time‐locked RFD and (2) in RFD_peak_ in burst‐like isometric knee extensions.

## 2 MATERIALS AND METHODS

### 2.1 Participants

Twenty‐four (five female) healthy adults volunteered (mean ± SD.; 25 ± 2 years; 71.2 ± 10.6 kg; 174 ± 8 cm) for this study. Participants were physically active practicing leisure physical activity for at least 2 times per week. Exclusion criteria were any previous history of neuromuscular disorders or lower limb injury in the previous six months. All the participants were informed about the testing procedure and provided written informed consent prior to their participation in this study, which was approved by the Ethical Advisory Committee (University of Verona—approval no: 13.R1/2021) and performed in accordance with the Helsinki Declaration.

Participants visited the laboratory only once and avoided strenuous exercise for 24 h and caffeine for 6 h before the experimental session. The experimental session was divided into two parts. The first part was constituted of a warm‐up and familiarization with real‐time visual feedback, which continued until the participant was able to perform five consecutives explosive contractions without countermovement or holding phase. The second part was constituted by the actual measurement, lasting 30 min. All measurements were taken from the participant's right lower limb (which was the dominant limb in 22 to 24 participants).

### 2.2 Experimental setup

Participants were seated on a custom‐made chair that allowed the assessment of the right knee extensors. Straps were fastened across the chest and hips to avoid lateral and frontal displacements. The participant's knee and hip were flexed at 90° from full extension. The strain gauge load cell (546QD‐ 220 kg; DSEurope, Milan, Italy) was positioned 2 cm above the malleolus perpendicular to the tibial alignment. To avoid pain and maintain structural stiffness, a standard hard shin protector was placed between the thrust surface and the tibia.

### 2.3 Voluntary contractions

Participants performed a series of 10 warm‐up contractions at progressively higher levels of force before completing two maximal voluntary contractions, with 2 min of rest in between. Participants were instructed to push as hard as possible for 5 s, and they received standardized strong verbal encouragement. Participants also received visual real‐time feedback regarding the force response through a screen placed in their line of sight.

**FIGURE 1:**
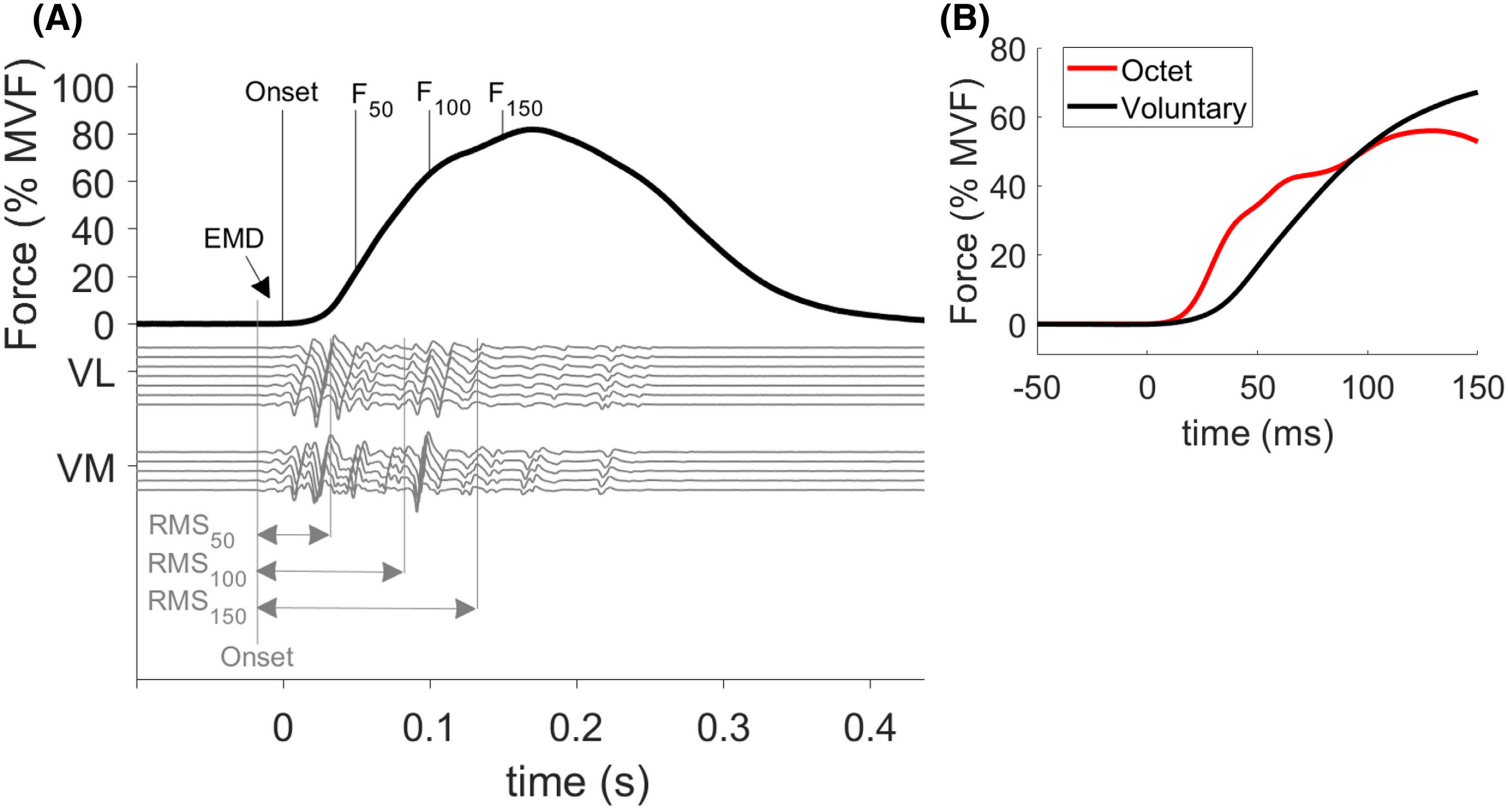
(A) Example recordings of force (black) and HDsEMG channels (gray) during a burst‐like (i.e., Gaussian‐shaped) explosive isometric voluntary contraction. It should be noted that the duration is approximately 200 ms and that the EMG onset starts a few milliseconds before the force onset. The force expressed at 50 (F_50_), 100 (F_100_), and 150 ms (F_150_) after the onset of contraction were considered the dependent variables in the stepwise multiple regression models. Only one representative column of each matrix of electrodes are reported for vastus lateralis (VL) and medialis (VM) muscles. The time periods over which the EMG variables were calculated (RMS and muscle fiber conduction velocity) are highlighted by the gray arrows. (B) Representative example of the first 150 ms of the voluntary (black) and electrically evoked octet (red) contractions

After 3 min of rest, the participants performed 10 explosive contractions (brief pulses) interspersed by 15 s of rest. The contractions were characterized by a short active phase (lasting ≈ 200 ms) and by avoiding any holding phase, resulting in a burst‐like shape (Figure 1A). Participants were instructed to push “as fast and as hard as possible”.^25^ A visual line on the screen depicted 80% of MVF with a target error of ±10%^10^ during the contractions, and participants were instructed to achieve a peak force above this level during each explosive contraction.

The analog force signal was amplified (gain 150) and sampled at 2048 Hz with an external analog‐to‐digital converter (QUATTROCENTO; OT Bioelettronica, Turin, Italy). and the data were recorded with the software OT BioLab (OT Bioelettronica, Turin, Italy) and analyzed with MATLAB (v. 2020b, The Mathworks, Natick, MA).

### 2.4 Electrically evoked twitch and octet contractions

Electrical stimuli were delivered via percutaneous stimulation of the femoral nerve in the femoral triangle via a constant‐current, variable‐voltage stimulator (DS7AH; Digitimer Ltd, Welwyn Garden City, UK). The anode (50 x 90 mm) was placed over the greater trochanter and the cathode (Ø = 32 mm) was placed within the femoral triangle, near the femoral nerve. We adopted square‐wave pulses (0.2 ms in duration) with maximal voltage of 400 V to elicit either single or octet‐train contractions (eight pulses at 300 Hz). The adoption of octets has been utilized to determine the maximal capacity of the muscle–tendon unit to produce explosive force,^13^ see Figure 1B. While this procedure was painful for the participants, we did not have any drop‐out because of this.

A series of incremental (starting from 20 mA and increasing by 20 mA at each step) single stimuli were introduced until there was a plateau in the M‐wave amplitude response,^10^ which was visually evaluated. One representative channel of the 64 available for the vastus lateralis (VL) was adopted for this aim (see High‐Density Surface Electromyography paragraph). Another series of incremental stimuli was used to find the force signal plateau for octet stimulation.^13^ During all stimulations, the experimenter pressed with his hand on the anode to bring it closer to the cathode and to obtain a better response. The intensity plateau values (range: 120–400 mA) for single and octets were each increased by 20% to ensure supramaximal stimulation. Two single twitches and two octet contractions (each one interspersed by 10 s) were delivered.

### 2.5 High‐density surface electromyography

High‐density surface electromyography (HDsEMG) signals were recorded from the vastus lateralis (VL) and vastus medialis (VM) muscles in a single differential configuration using two matrices of 64 electrodes each (13 rows × 5 columns, 8 mm inter‐electrode distance, gold‐coated; model: GR08MM1305, OT Bioelettronica, Turin, Italy). The reference electrode (24 mm, model: CDE‐S. OT Bioelettronica, Turin, Italy) was placed on the patella of the same limb. Before the placement of the electrodes, the skin area was shaved and then was slightly abraded with abrasive paste and cleaned with water.^26^ To ensure proper electrode–skin contact, the electrode cavities of the matrix were filled with conductive paste (Spes‐Medica, Battipaglia, Italy). The electrode arrays were fixed with an extensible tape. The EMG signals were amplified (gain 150), sampled at 2048 Hz and converted to digital data with a 16‐bit A/D converter (Quattrocento; OT Bioelettronica, Turin, Italy). Signals, in single‐differential configuration, were visualized during acquisition and then stored on a personal computer using OT BioLab+ software version 1.5.5.0 (OT Bioelettronica, Turin, Italy) for further analysis.

### 2.6 Data analysis

#### 2.6.1 Force signals

The force signals were low‐pass‐filtered at 50 Hz using a fourth‐order zero‐lag Butterworth. MVF was defined as the highest force over the two maximal voluntary contractions. The onset of each voluntary and evoked contraction was visually assessed^27^ by the same researcher through a hand‐customized MATLAB code. In the case of countermovement, the contraction was discarded. Force was assessed at 50, 100, and 150 ms after the contraction's onset for voluntary (F_50_, F_100_, F_150_), evoked single (F_single_50_, F_single_100_, F_single_150_), and octet (F_octet_50_, F_octet_100_, F_octet_150_) stimuli. RFD_peak_ was calculated as the maximum of the first derivative of force overtime on the filtered signals. Out of 10 ballistic contractions, we discarded the contractions showing the highest and lowest RFD_peak_. Then, we averaged the force and EMG variables (see next paragraph) calculated from the remaining eight contractions to increase the precision of the estimates. Regarding the evoked contractions, we averaged the values calculated from the two single and two octet contractions.

### 2.7 High‐density surface electromyography

The EMG signals were band‐pass‐filtered at 30–450 Hz using a fourth‐order zero‐lag Butterworth filter prior to analysis. We first removed the EMG channels showing excessive noise or artifacts through visual analysis. Signals for each column of electrodes were visually inspected, and the four to eight (depending on columns) single‐differential HDsEMG signal channels with clear motor unit action potential propagation without shape change were chosen for the analysis. The average number of selected signals were 6 (from 4 to 8 depending on columns). The onset of each EMG signal of each contraction was visually selected^28^ by the same researcher through a hand‐customized MATLAB code. The RMS was calculated across all available channels, divided by the M‐wave amplitude (elicited by the single stimuli) calculated over the same electrodes. RMS was then averaged across VL and VM channels to obtain a single estimate calculated over the first 50, 100 and 150 ms from EMG onset (RMS_50_, RMS_100_, and RMS_150_).

MFCV was calculated using an algorithm that allows the estimation of conduction velocities from multichannel EMG signals in explosive burst‐like contractions.^29^ The algorithm provides highly accurate estimates over epochs as short as 50 ms. Therefore, the MFCV was calculated over the first 50, 100, and 150 ms from EMG onset (defined as MFCV_50_, MFCV_100_, and MFCV_150_) for the ballistic contractions. We also calculated the MFCV of the M‐wave over the same channels adopted for the voluntary contractions. Then, MFCV was also calculated in relative terms with the M‐wave conduction velocity (defined as MFCV_rel_50_, MFCV_rel _100_, and MFCV_rel _150_). Finally, the MFCV estimates of voluntary contractions and M‐wave were calculated over the five columns of electrodes (for each muscle) and then averaged.

### 2.8 Statistical analysis

Descriptive statistics are presented as mean ± SD. and coefficient of variation (COV, %) where needed. Statistical analyses were performed using JASP (Version 0.16), and statistical significance was set at *p* < 0.05. Preliminary analysis with Pearson's correlation between purely explosive voluntary force and individual predictor variables was performed. To answer the first experimental question, multiple stepwise linear regression between voluntary force calculated over successive 50 ms time periods (50, 100 and 150 ms) and predictor variables was performed to assess the influence of all the entered predictor variables simultaneously. Predictor variables were assessed at the identical time period of the relevant voluntary force measurement. For example, the possible predictors of F_50_ were F_octet_50_, F_single_50_, MFCV_rel _50_, RMS_50,_ and MVF. However, for evoked measures, which were of shorter duration, we always adopted the response at 50 ms (i.e., F_octet_50_ and F_single_50_). To answer the second experimental question, stepwise linear regression analysis between RFD_peak_ and predictor variables was performed. To make it clearer: the mechanical variables (MVF, F_50_, F_100_, and F_150_) were entered as dependent variables in absolute terms. To avoid the effect of some confounding factors (such as for example subcutaneous adipose tissue), we normalized the EMG variables for the M‐wave characteristics: RMS was divided by the M‐wave amplitude; MFCV was divided by the MFCV of M‐wave.

## 3 RESULTS

### 3.1 Interindividual variability

The MVF of knee extensors was 424 ± 91 N with a COV of 21%, ideally in line with the data reported by Folland and colleagues.^10^ The COV of variables assessed during the voluntary contractions is reported in Table 1. Briefly, the COV of force ranged from 37% at 50 ms to 23% at 150 ms. The COV of RMS (normalized by M‐wave amplitude) ranged from 43% at 50 ms to 23% at 150 ms. The COV of MFCV (normalized by M‐wave conduction velocity) ranged from 10% at 50 ms to 11% at 150 ms. The COV of absolute MFCV ranged from 12% at 50 ms to 15% at 150 ms. The peak‐to‐peak amplitude of M‐Wave was 3801 ± 1048 mV and the COV was 28%. The COV of octet forces ranged from 23% at 50 ms to 30% at 150 ms. The COV of maximal M‐wave was 28%. To confirm the brevity of the explosive contraction, through calculation, we determined that the time to peak force was 180 ± 30 ms with a COV of 16%.

**TABLE 1:** Mean, standard deviation (SD), and coefficient of variation (COV, %) assessed at 50, 100, and 150 ms for RMS (mV), MFCV (m/s) in absolute (MFCV_abs), and relative (normalized for M‐Wave; MFCV_rel) terms, force, twitch, and octet

	RMS			RMS (%)			MFCV_abs			MFCV_rel			Force_abs			Force (%)			Twitch			Octet		
	Mean	SD	COV	Mean	SD	COV	Mean	SD	COV	Mean	SD	COV	Mean	SD	COV	Mean	SD	COV	Mean	SD	COV	Mean	SD	COV
50	0.037	0.016	43	1.098	0.708	64	4.7	0.6	12	1.074	0.103	10	72	27	37	17.5	6.9	39	71.9	22	31	139.2	32.1	23
100	0.063	0.014	22	1.847	0.888	48	4.9	0.6	13	1.113	0.107	10	223	49	22	53.2	7.7	14	78.1	28.9	37	205.9	60.5	29
150	0.064	0.015	23	1.871	0.946	51	5.3	0.8	15	1.197	0.138	12	287	66	23	67.9	8.2	12	43.1	18.9	44	216.3	65.5	30

*Note*: RMS (mV) is reported as the mean of the values assessed from VL and VM. Force (N), twitch (N), and octet (N) are reported in absolute terms. RMS (%) is expressed as percentage of maximal M‐wave whereas Force (%) as percentage of MVF.

Pearson's correlation coefficients between predictor variables and voluntary force are reported in Table 2. Briefly, F_50_ was significantly positively correlated only with RMS_50_, while F_100_ and F_150_ were positively correlated with MVF, single twitch, and octets.

**TABLE 2:** Pearson's correlation coefficients between predictor variables and voluntary explosive force of the knee extensors during the first, 50 (F_50_), 100 (F_100_), and 150 ms (F_150_) of contraction (*n* = 24)

	F_50_	F_100_	F_150_	RFD_peak_
MVF	0.202	0.775***	0.851***	0.734***
RMS	0.624**	−0.024	0.084	0.257
MFCV_rel	−0.322	−0.122	0.053	−0.033
MFCV_abs	−0.195	−0.249	−0.131	−0.140
TWITCH	0.173	0.681***	0.723***	0.463*
OCTET	0.229	0.813***	0.829***	0.638***

*Note*: All the variables are considered in absolute terms, except for MFCV_rel, which is expressed in relative terms with M‐wave. RMS, MFCV_abs, and MFCV_rel are considered as mean values assessed in VL and VM.

* *p* < 0.05;

** *p* < 0.01;

*** *p* < 0.001.

### 3.2 Determinants of explosive absolute force

**FIGURE 2:**
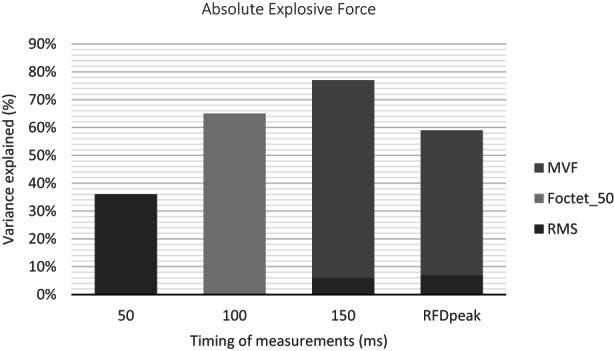
Determinants of RFD_peak_ and absolute voluntary purely explosive force of the knee extensors during the first 150 ms of contraction. Predictor variables that independently explained a significant proportion of the total variance assessed with stepwise multiple linear regressions are shown

The multiple regression analysis showed that the total variance in explosive force explained by the predictor variables increased throughout the contraction from 36% at 50 ms to 65% at 100 ms and to 77% at 150 ms, but the contribution of specific predictor variables changed over the contraction duration. Figure 2 represents the variance accounted for by predictors from 50 to 150 ms of contraction. Briefly, F_50_ was explained by only RMS_50_ (*R* ^2^ = 0.361, *p* = 0.001). F_100_ was explained by F_octet_50_ (*R* ^2^ = 0.646, *p* < 0.001). F_150_ was explained by MVF (*R* ^2^ = 0.711, *p* < 0.001) and F_octet_50_ (*R* ^2^ = 0.061, *p* = 0.016).

### 3.3 RFD_peak_

The average RFD_peak_ was 4752 ± 1185 N∙s^−1^ with a COV of 25%. The time to peak RFD was 60 ± 10 ms with a COV of 21%. Pearson's correlation analysis showed that RFD_peak_ positively correlated with MVF, single twitch, and octets (see Table 2). The multiple regression analysis showed that the RFD_peak_ was explained by MVF (*R* ^2^ = 0.518, *p* < 0.001) and by RMS_50_ (*R* ^2^ = 0.074, *p* = 0.036), accounting for a total of 59.2% of the variance.

## 4 DISCUSSION

We analyzed neural and contractile determinants of burst‐like explosive contractions in the knee extensors of healthy young adults through the application of electrically evoked single twitches, octets, and HDsEMG. The force production was related to different neuromuscular factors that changed through the contraction timing from onset: while the early RFD (F_50_) was correlated only to RMS_50_, late RFD (F_100_ and F_150_) was more correlated to F_octet_50_ and MVF. The multiple regression analysis showed that 36% of the variance of F_50_ was explained by RMS_50_; 65% of the variance of F_100_ was explained by F_octet_50_; 71% of the variance of F_150_ was explained by MVF and another 6% by F_octet_50_. For the RFD_peak_ (which occurred, on average, 60 ms after force onset) 52% was explained by MVF and another 7% by RMS_50_. MFCV calculated from HDsEMG did not contribute to explaining the variance of any variables considered.

This is the first study that has inspected the neural and contractile determinants of burst‐like explosive isometric contractions. This is particularly relevant because while, in real‐life, the muscle activation profiles are Gaussian‐shaped,^21^, ^22^ that is, without holding the phase of maximal contraction. Differently, previous studies adopted explosive contractions with a holding phase (of at least 1 s).^9^, ^10^, ^11^, ^15^, ^18^ Therefore, while our results are mostly in line with previous works adopting contractions with a holding phase,^9^, ^10^, ^11^, ^30^ in the present study we refine the determinants of RFD on explosive contraction of Gaussian‐shaped and shorter (<200 ms) duration.

The main determinant of early RFD was the muscle excitation over the first 50 ms of contraction. This finding is in line with previous works^11^, ^27^ and it supports the hypothesis that the physiological variation in the rate by which motor units are recruited during ballistic contractions is the main determinant of the variability in RFD across individuals.^14^, ^31^ For this reason, athletes that showed greater RFD also showed greater muscle excitation over the first 50 ms of an explosive contraction compared with non‐trained adults.^27^ Indeed, after a period of ballistic training, the motor unit discharge rates tend to increase and early RFD depends on the motor unit discharge rate.^32^ As a methodological note, we calculated the RMS over dozens of electrodes from HD‐EMG; we consider this measure more reliable than previous works adopting only couples of electrodes. Furthermore, as we divided the RMS calculated from each channel by the M‐wave amplitude of each channel, the muscle excitation calculated in this study can be considered more valid than that calculated using absolute RMS.

Late RFD (≥100 ms) was correlated to both contractile properties (F_single_50_ and F_octet_50_) and MVF (see Table 2), with some differences regarding the determinants of force at 100 and 150 ms. Force production at 100 ms was mostly explained by the contractile properties (F_octet_50_), and this is in line with Folland et al,^10^ which found that F_octet_50_ was the primary determinant of voluntary RFD assessed at 50–100 ms, accounting for 68% of variance. Quadriceps muscle volume can also influence contractile properties (measured with the evoked octet) and consequently late RFD.^15^ Contractile properties are also dependent on fiber type composition^33^ and muscle‐tendon unit stiffness,^17^, ^34^ which has been related to RFD in the first 50–100 ms. Force at 150 ms was in part explained by MVF, and this confirms the results of Cossich and Maffiuletti,^11^ who found that the relationship between MVF and RFD was stronger with the late than early RFD. This is also in line with Andersen and Aagard^9^ as they found that voluntary RFD became increasingly dependent on MVF and less dependent on evoked twitch as the time from the onset of contraction increased.

Unexpectedly, MFCV did not correlate with force production in any time interval, from 50 to 150 ms (see Table 2). Analysis of MFCV has been suggested to provide an indirect assessment of the properties of active motor units.^35^ It is well known that the MFCV is dependent on fiber diameter.^35^, ^36^ Therefore, MFCV is considered a size principle parameter^35^ since the muscle fibers of high‐threshold motor units have greater diameters than those of lower‐threshold motor units.^35^ For this reason, we expected that participants able to more rapidly recruit larger motor units would be able to exert a higher RFD. Indeed, Del Vecchio et al,^30^ found a positive correlation between the early RFD (50 ms) of elbow flexion and MFCV of biceps brachii. Besides the investigation of different muscles (VM and VL in our study), we are unable to identify any methodological difference that could explain the disagreement between our study and that of Del Vecchio et al,^30^ as we adopted the same algorithm to calculate MFCV over a short period of time.^29^ To account for differences in muscle fiber diameter, we normalized MFCV to M‐wave MFCV, but in this case, as well, we did not find any correlation with force production. The present results do not corroborate the usefulness of analyzing MFCV in explosive contraction. However, we could not exclude that the muscle of interest and the sample characteristics may play a role in the relationship between MFCV and RFD.

Despite its wide popularity,^23^, ^24^ the physiological determinants of RFD_peak_ have been little studied. As the time to peak RFD was, on average, 60 ± 10 ms, we expected that the determinants of RFD_peak_ would have been more similar those of F_50_ than F_100_ and F_150_. Contrary to our expectations, the main determinant of RFD_peak_ was MVF (see Figure 2), while that of F_50_ was RMS_50_. This means that the highest steepness of the force–time curve is related to the highest level of voluntary force. The analysis of RFD_peak_ is easy to perform because it does not require the identification of the onset of the muscle contraction, as occurs in the time‐locked analysis of RFD. However, the present results suggest that RFD_peak_ is in part related to MVF and therefore may provide similar information to this parameter. Conversely, time‐locked analysis of RFD, especially in the first 50 ms, may provide further insight into the neuromuscular characteristics of explosive contractions.

Regarding the limitations of the present study, in addition to the non‐homogeneity of the sample with only three female participants, we should consider that many physiological variables such as muscle size and architecture,^11^, ^15^ muscle–tendon stiffness,^37^ belly‐gearing,^34^ motor units recruitment speed, and discharge rate^30^ played a role and were not investigated. In addition, we focused only on the initial milliseconds of force contractions, and we did not assess voluntary activation over the plateau phase of a maximal voluntary contraction through. Furthermore, we only focused on isometric contraction^38^; therefore, the present results might not be valid for dynamic contractions.

### 4.1 Perspectives

The analysis of Gaussian‐shaped muscle contractions is scarce compared to that of other forms of explosive contractions with some sort of holding phase. In accordance with Folland et al,^10^ the relative contribution of different determinants changed during the phase of contraction, and this finding may be useful in the design of interventions to improve explosive force production.

The time intervals for RFD calculation should be better justified and the distinction between “early” and “late” RFD should be based on physiological relevance. In the present study, we found that the force production at 100 ms has determinants similar to that at 150 ms (i.e., both time windows include peripheral factors); therefore, the RFD calculated over 100 ms of contraction should be considered “late RFD,” while the RFD in the first 50 ms should be considered “early RFD.” Moreover, the fact that the RFD_peak_ is in part explained by MVF must be taken into account in experimental studies as it may provide similar information to MVF without sufficiently differentiating from it. Therefore, the analysis of time‐locked intervals is preferred as it provides more insights into the neuromuscular characteristics of explosive contractions.

## 5 CONCLUSIONS

The force production of burst‐like explosive knee extensor contractions was regulated by different neuromuscular factors that changed throughout the contraction duration. The main determinant of force production in the first 50 ms (early RFD) was the muscle excitation calculated over the vastus medialis and lateralis muscles. The main determinants of force production in the first 100 and 150 ms (late RFD) were contractile properties and MVF. In particular, contractile properties were more determinant for force production in the first 100 ms, while MVF was more determinant for force production in the first 150 ms. The main determinant of the peak of RFD was the MVF, which means that RFD_peak_ as an index of explosive contraction may not provide more information to differentiate between participants.

## AUTHOR CONTRIBUTIONS

Samuel D'Emanuele, Gennaro Boccia, Federico Schena, and Cantor Tarperi conceived the study. Samuel D'Emanuele collected the data. Cantor Tarperi and Federico Schena supervised the data collection. Gennaro Boccia analyzed the data. Samuel D'Emanuele and Gennaro Boccia performed the statistical analysis. Samuel D'Emanuele and Gennaro Boccia wrote the draft, and Federico Schena and Cantor Tarperi revised the draft.

## CONFLICT OF INTEREST

No conflicts of interest, financial or otherwise, are declared by the authors.

## Data Availability

The data that support the findings of this study are available from the corresponding author upon reasonable request.
